# Medical and Physical Intelligence

**Published:** 1802-08-01

**Authors:** 


### MEDICAL AND PHYSICAL INTELLIGENCE.

### [ FOREIGN AND DOMESTIC. ]

### *Observations on the Effects of the Carbonous Gas (Gaz Carboneux) in Animal Oeconomy, by Cit.* CHAUSSIER.

About twenty years ago, it was generally believed that ſaltpetre being melted on burning coals furniſhed vital air, and thus purified the atmoſphere; Cit. *Chaussier*, however diſcovered, that this proceeding was very far from being of any uſe, but rather dangerous, as it produced an irreſpirable gas, which is inſoluble in water, and heavier than what is properly called *inflammable gas*. An analyſis of that gas has ſince that time been made by *Guyton, Desormes*, and *Clement*, according to which it conſiſts of gas carbonic acid and of carbonous gas; the firſt of which contains in 100 parts about 27 or 28 parts of carbon, while the latter had in 100 parts from 46 to 52 of carbon. With this carbonous gas, Cit. *Chaussier* has made ſeveral experiments on living animals, and on freſh veinous blood; and in order to obtain a better knowledge of its particular action, he has repeated at the ſame time the experiments with the other irreſpirable gaziform fluids. The reſults of all theſe experiments are the following:

In *pure hydrogen gas*, the aſphyxia is ſlowly produced; the blood and other parts receive a browniſh colour.

In *sulphurous hydrogen gas*, the aſphyxia is ſuddenly brought on; the blood, the liver, and all other parts take a black colour.

In *carbonised hydrogen gas*, the aſphyxia enſues leſs quickly than in the gas carbonic acid, but more rapidly than in pure hydrogen gas; the blood and the other parts of the body obtained a vermilion colour.

In the gas *carbonic acid* an aſphyxia ſupervenes in a few minutes; and in conſequence of the convulſive efforts to reſpire, the muſcles are quickly deprived of their irritability; the blood coagulates a little, and receives a dark colour. It frequently happens, that the lungs will not ſwim on the ſurface of water, but go down to the bottom.

In the *carbonous gas*, the aſphyxia enſues gradually, the muſcles remain longer irritable, and the blood and other parts receive a ſcarlet colour.

It appears from theſe experiments, that the gaſſes which contain carbon impart to the blood a vermilion colour, analogous to that which it obtains by abſorbing oxygen.

### *Preparation of the Naphtha Aceti Martialis.*

Diſſolve pure iron filings in pure muriatic acid, and when the ſolution is perfectly performed, add ſmall portions of pure nitric acid, acid, which continue to do till nitrous gas is no longer difengaged, and till the iron is perfectly oxydated. Having diluted the folution with water, impregnate it with the lye of kali or natron. The precipitation thus obtained muft be properly edulcorated and dried in the air, till it appears in form of a fomewhat moift and brittle mafs. In this ftate the ferrugineous precipitation is added by fmall portions to the acetic acid, and frequently ftirred with a glafs tube, which muft be continued till the acid is perfectly faturated. Nine ounces of this concentrated brown folution are mixed with one ounce of æther aceticus and fpiritus vini alcoholifatus, and the whole mixture preferved under the above appellation.—— Another mode of preparing this Naphtha, recommended by Mr. Fittner, is the following: One ounce of well dried fulphat of iron, or iron vitriol calcinated to perfect whitenefs, and one ounce and a half of acetati of kali, or terra foliata tartari, are ground in a ftone mortar and intimately mixed: this mixture having been expofed for a few days to the open air, to make it perfectly dry, is diffolved in a mixture of fix ounces of diftilled water and the fame quantity of the very beft alkohol, but without employing any heat. The liquor being feparated from the refiduums by the filtrum, fix drachms of æther aceticus and 3 drachms of acetic acid are added, and the filtrum wafhed with a mixture of two parts water and one of alkohol, till the weight of the filtrated liquor (including the acetic acid and the æther aceticus) amounts to 9 ounces, which ought to be preferved in well clofed glaffes.

---

CIT. BERTHOLLET has prefented to the National Inftitute a Memoir on the fulminating mercury, a preparation firft difcovered by Mr. Howard, which, though it poffeffes the property of fulminating, is produced by a proceeding different from that in which the aurum and argentum fulminans are obtained; becaufe the fulminating mercury is produced by the ebullition of the nitrat of mercury in alkohol, whereby it is depofited in form of a grey or white powder. The analyfis of this fubftance being the only way of explaining as well the production of it as its properties, this, confequently, ought particularly to engage the attention of chemifts. The author of this curious difcovery had concluded from his experiments, that the fulminating mercury was compofed in 100 parts, 21,28 of oxalic acid, 64,72 of mercury, and 14 of etherated nitrous gas, and of a furplus of oxygen. On confidering, however, this compofition, we muft confefs, that it does not fufficiently account for the violent explofions of that preparation; and for this reafon Cit. Berthollet undertook a new analyfis, the refults of which differ extremely from thofe given by Mr. Howard. The liquor, fwimming on the preparation, contains mercury, and yields, with lime, a black precipitation in the fame manner as is the cafe with mercurial folutions that contain ammonia, the exhalations of which are here fenfibly perceived. The powder itfelf difengages ammonia when treated with potafh, but no oxalic acid could be be traced in it by means of the fame alkali. The fulminating mercury diffolves in muriatic acid, but after having precipitated from this folution the metal by means of the hydrofulphur of potafh, the muriat of lime did not produce any precipitation in the liquor, as would have been the cafe if it had been oxalat of mercury. A fimilar folution yielded by diftillation minute needles, which were nothing but a muriat of mercury and ammonia. Cit. Berthollet concludes from his experiments, that the fulminating mercury contains no oxalic acid, but ammonia with the oxyd of mercury. Thefe, however, are not its only conftituent particles, as in the aurum and argentum fulminans, which appears from its decompofition by diluted fulphuric acid. The fulminating mercury is changed by this acid into a white powder, which does not any longer occafion detonation. This powder Mr. Howard took for an oxalat of mercury, whereas Berthollet proves it to be a fweet fulphat of mercury. The action of the fulphuric acid difengages at the fame time a gas, which for the moft part confifts of carbonic acid, about the twelfth part of it only being hydrogen gas oxicarbonated. The fulminating mercury contains, confequently, a fubftance which is eafy to be decompofed; at leaft Cit. Berthollet could not feparate it, without at the fame time decompofing it, and he confiders it in its nature as approaching to alkohol. The metal appears to be in the fame ftate of oxydation in the fulminating mercury as in corrofive mercury; but it is defoxydated by the decompofition which that alkoholic fubftance experiences by means of the fulphuric acid, fo as to form a fweet fulphat with the faid acid.

---

VAUQUELIN, *on the Nature of the Earth which is eaten by the Inhabitants of New Caledonia.*

Cit. Labillardiere, on his voyage, had made the curious obfervation, that the inhabitants of New Caledonia, when preffed by hunger, devour a confiderable quantity of a greenifh fteatites, (foap ftone) which is very tender and friable. It may hence be eafily conceived, how that horrid cuftom of eating the prifoners of war could be introduced amongft thefe favages, who in time of famine, have recourfe for appeafing a hungry ftomach to a terreous fubftance, which only diftends the ftomach and the inteftines, and has no other alimentary quality but that of being light and friable. Cit. Vauquelin, curious to know the nature of this earth, and whether it contained any thing nutritive, made an analyfis of fome fpecimens which he had received from Labillardiere. This earth is foft to the touch, and formed of fmall threads that are eafily divifible: It becomes red by fire, whereby it lofes $\frac{4}{100}$ of its weight. It is compofed of 37 parts of pure magnefia.
36 of filiceous earth.
17 of oxyd.
3 or 4 of water.
2 or 3 of lime and copper.

It

It contains therefore not a ſingle nutritive particle, and muſt only be conſidered as a ſort of ballaſt, or a mechanic expedient to aſſuage the pains cauſed by hunger.

---

At the annual Diſtrict Meeting of the Benevolent Medical Society of Eſſex and Herts, held at Hatfield in Herts, May 3, 1802: The underſigned being ſtrongly impreſſed with the invaluable advantages that the public have derived from the introduction of the Vaccine Diſeaſe, by Dr. Jenner, as a ſubſtitute for the Small-pox, are deſirous of preſenting their thanks to him for the liberality and indefatigable induſtry with which he has made it public.

JAMES PENROSE,

JOHN KINGSTON,

JOHN WINKFIELD,

SMYTH CHURCHILL,

ROBERT OLDMEADOW,

JOHN DARBY,

C. E. LUCAS,

E. HARROLD,

THOMAS COLBECK.

---

Mr. WILKINSON, Surgeon, of Sunderland, has in hand a work preparing for the preſs, to be entitled Experiments and Obſervations on the Cortex Salicis Latifoliæ, to be illuſtrated by an accurate coloured plate of a branch of the tree and its bark, in a recent and likewiſe in a dried ſtate.

---

